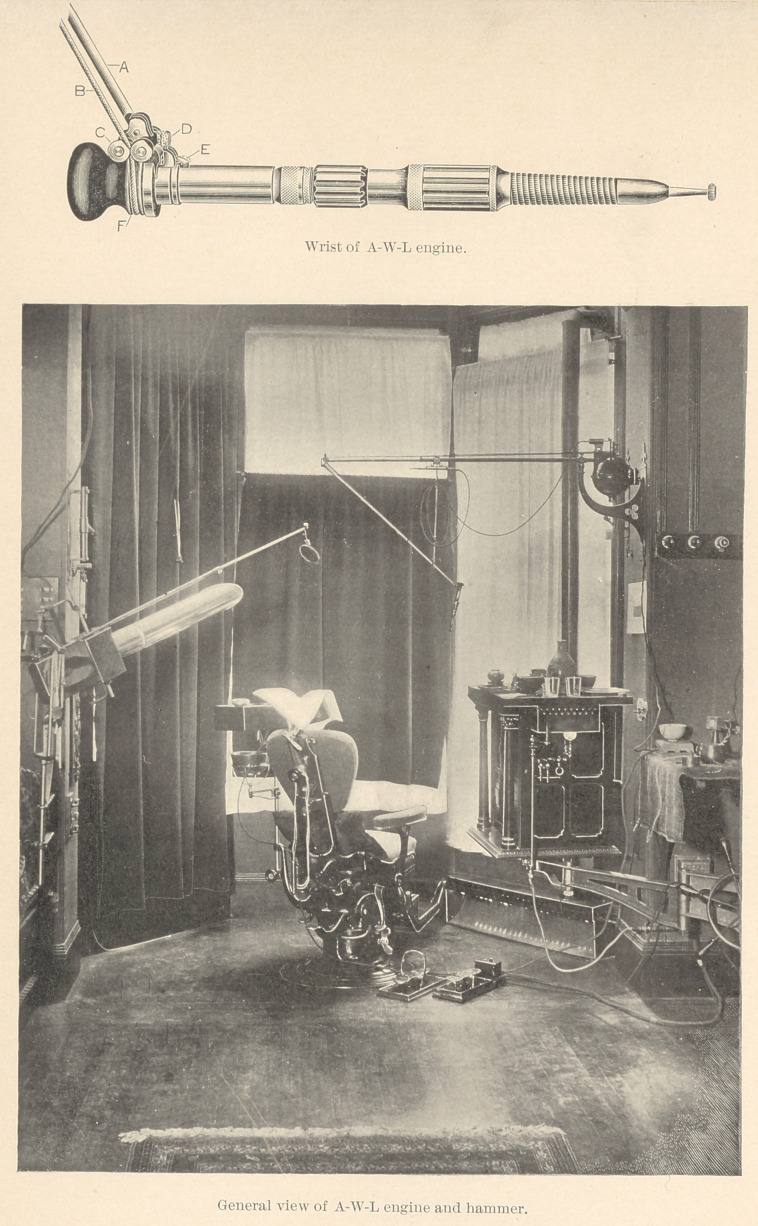# Dental Notes

**Published:** 1900-04

**Authors:** William Rollins

**Affiliations:** Boston, Mass.


### DENTAL NOTES.

BY WILLIAM ROLLINS, BOSTON, MASS.

### NOTE V. THE A-W-L ENGINE.

My engine was the first in which a wrist-joint was placed in its normal position, that of suspension from the forearm. Taken in connection with the A-W-L hammer, it is a convenient piece of mechanism. The half-tone shows its arrangement in repose. The motor is large and slow-running. My patients do not like the jar of rapidly moving grinding wheels, nor rapid blows from the hammer. A thousand revolutions is enough for a bur and six hundred blows a minute enough for a hammer. Less speeds can be had by means of a Berry foot-switch, which has served me, without cost for repairs, during a number of years. No attempt, therefore, is made to show another type of foot-switch, nor is the hand-piece of my design. It was made by my friend, Frank K. Hesse. The engine moves in altitude and azimuth on ball bearings, balancing with a sliding motion. The wrist-joint is interesting for its age and simplicity. I showed the principle in a power-driven engine twenty-five years ago, yet wrist-joints with foolish and unnecessary parts have held the field since. Two things made this joint practical: the rotation of the hand-piece on its long axis and the relative position of the axes of the guides to the axis on which the hand-piece turns on its sustaining arm. The latter prevented the belt from getting tighter when the hand-piece was not at a right angle with the forearm.

Explanation of the cuts: Fig. 1 gives the general view of the motor end. Either the hammer or the engine can be worked separately by means of the projecting pins. Fig. 2 gives the details of the wrist-joint and lock for the revolving hand-piece.

DESCRIPTION OF FIGURE SHOWING MOTOR AND SUPPORT.

A, wrought-iron support.

B, Holtzer-Cabot one-sixth horse-power motor.

C, ball bearings and vaseline cup for lower end of armature shaft.

D, iron tube holding horizontal arm.

E, balancing sleeve with clamp, F.

G, head with ball bearings for movement in vertical arc.

H, covers to ball bearings.

I, pin for ball bearing for movement in horizontal arc.

J, air-pump for hammer.

K, wide bearing pulley for crank-pin to hammer. This wide bearing prevents noise.

L, bearing pulley for cord to hand-piece. There should be three grooves here for different speeds.

M and N, stopping and starting pins to hammer and engine to enable both to go together or either alone.

O, vaseline cup.

R, rubber tube to hammer.

S, cord to hand-piece.

T, wires to main and switch.

Scale 23-84.

The A-W-L engine.

William Rollins

Wrist of A-W-L engine.

General view of A-W-L engine and hammer.

DESCRIPTION OF FIGURE OF WRIST JOINT.

A, forearm.
B, cord.
C, guides.
D, release for hand-piece.
E, catch holding hand-piece and allowing it to revolve.

## Figures and Tables

**Figure f1:**
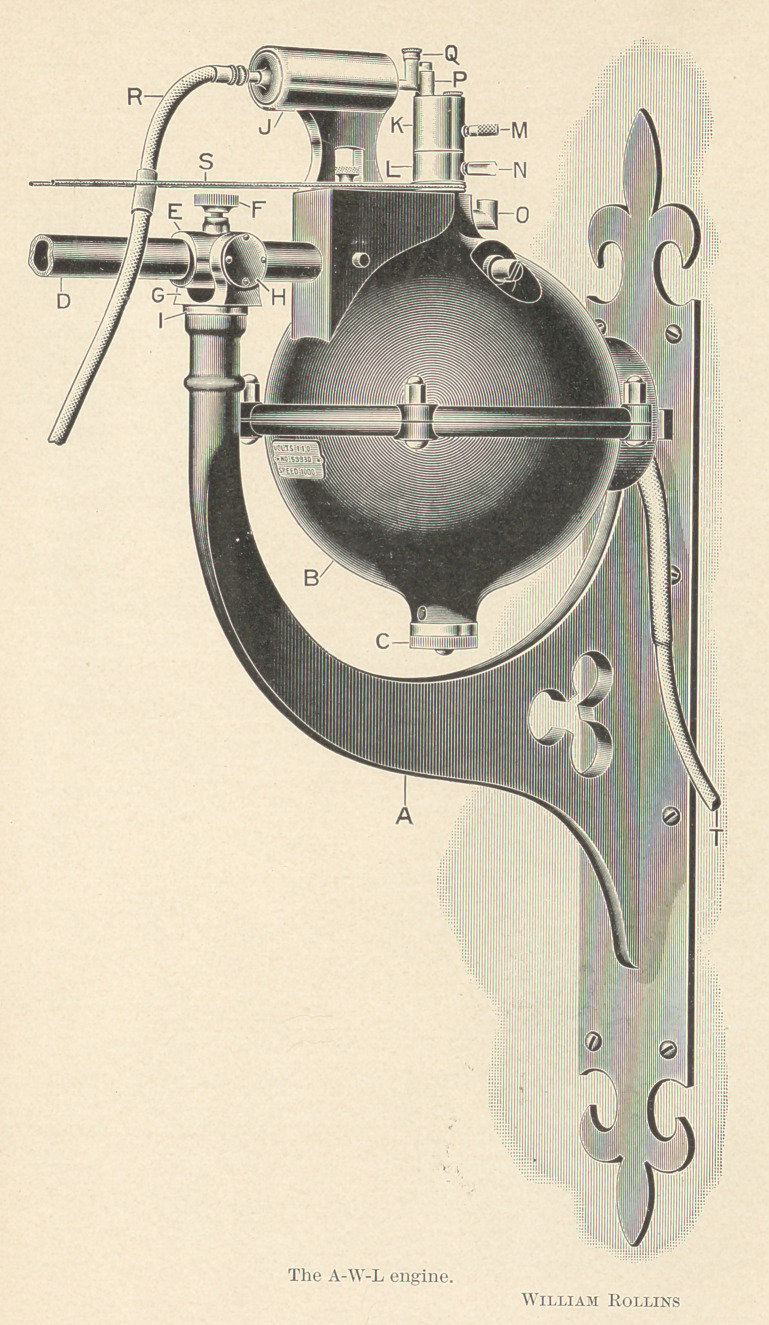


**Figure f2:**